# A Review of PZT Patches Applications in Submerged Systems

**DOI:** 10.3390/s18072251

**Published:** 2018-07-12

**Authors:** Alexandre Presas, Yongyao Luo, Zhengwei Wang, David Valentin, Mònica Egusquiza

**Affiliations:** 1 Department of Energy and Power Engineering, Tsinghua University, Beijing 100084, China; alexpresas@tsinghua.edu.cn (A.P.); luoyy@tsinghua.edu.cn (Y.L.); 2 Center for Industrial Diagnostics and Fluid Dynamics (CDIF), Polytechnic University of Catalonia (UPC), Av. Diagonal, 647, ETSEIB, 08028 Barcelona, Spain; david.valentin@upc.edu (D.V.); monica.egusquiza@upc.edu (M.E.)

**Keywords:** PZT, submerged system, modal analysis, active vibration control, sound cancellation, AFM, energy harvesting

## Abstract

Submerged systems are found in many engineering, biological, and medicinal applications. For such systems, due to the particular environmental conditions and working medium, the research on the mechanical and structural properties at every scale (from macroscopic to nanoscopic), and the control of the system dynamics and induced effects become very difficult tasks. For such purposes in submerged systems, piezoelectric patches (PZTp), which are light, small and economic, have been proved to be a very good solution. PZTp have been recently used as sensors/actuators for applications such as modal analysis, active sound and vibration control, energy harvesting and atomic force microscopes in submerged systems. As a consequence, in these applications, newly developed transducers based on PZTp have become the most used ones, which has improved the state of the art and methods used in these fields. This review paper carefully analyzes and summarizes these applications particularized to submerged structures and shows the most relevant results and findings, which have been obtained thanks to the use of PZTp.

## 1. Introduction

Submerged systems and submerged structures are typically found in many applications including marine systems, naval systems, hydropower energy, biological systems, and medicine. These systems have the particular characteristic that they have a limited accessibility and, therefore, the research of their properties in every scale (from macroscale to nanoscale) and the control of the system dynamics are especially difficult and require from specific techniques. Typical applications in submerged systems that involve the research of mechanical and structural properties or control of the system dynamics are as follows: modal analysis, active vibration control, sound cancellation, atomic force microscopes, and energy harvesting. All these applications, which are studied and reviewed in the present paper, have the common characteristic that they need sensors and/or actuators installed in a system surrounded by a heavy fluid. As a consequence, the installation and use of transducers is much more complicated than in non-submerged systems as these systems are sometimes inaccessible, confined, withstand high flow velocities, fluid forces, and pressure fluctuations. Furthermore, the electronic parts of these transducers have to be protected from the surrounding fluid.

For these applications in submerged systems, one of the most used transducers (sensors & actuators) are the PZT patches (PZTp), which are based on the piezoelectric effect. In the following text we will refer to PZTp as a transducer made of PZT material, with a very small thickness (usually less than one millimeter) compared to the other main dimensions of the transducer. PZT patches are thin, light compared to other sensors/actuators and they are normally attached to a structure, although in some applications they can be used freely. The piezoelectric effect is a well-known property or capacity of some kinds of materials to exchange a mechanical deformation into electrical energy and vice versa. Although this effect was discovered more than one century ago (Curie, 1888), in the last two decades, due to the improvement of material science, PZTp of different sizes, shapes, and mechanical/electrical capacities have become widely used transducers in many applications.

This paper focuses on the most typical applications in submerged systems, where PZTp have been successfully used. It is shown that PZTp are the most preferred transducers in most of these applications and in some cases the sole option which has been practically implemented. Furthermore, this paper briefly presents and summarizes the main results and findings obtained in these applications, thanks to the use of PZTp. This review pretends to be a useful work for future research into submerged systems in applications related to modal analysis, vibration control, sound elimination, micro/nano research, and energy harvesting.

## 2. Constitutive Equations of Generic PZT Transducers

PZT transducers are made of piezoelectric materials that have the capacity to convert mechanical deformation into an electrical potential and vice versa, which is called direct piezoelectric effect and inverse piezoelectric effect respectively. This effect was first discovered by Pierre and Jacques Curie in 1880. The piezoelectric effect is anisotropic and can be only exhibited by materials with a crystal structure without a center of symmetry such as some ceramics below a certain temperature called Curie temperature. Such materials have electric dipoles randomly orientated that can be aligned along a poling direction with a poling process [1]. After this process these materials become permanently piezoelectric (if the temperature does not exceed Curie temperature or the electric field applied is too intensive), with the capacity to convert mechanical energy into electrical energy and vice versa. Therefore, they can be used as transducers for sensing or actuating purposes.

The relationship between mechanical and electrical energy in a PZT transducer is expressed by its constitutive equations, which have been presented in many studies [1,2,3]. Here we briefly present and discuss the constitutive equations of a transducer made of a one-dimensional piezoelectric material assuming that the electric field is parallel to the poling direction (see [1] for a more general cases). Let us assume a transducer is formed by a stack of *n* sheets with the same thickness and cross section (collocated in serie), with the same electric field between electrodes, and with electrical and mechanical quantities uniformly distributed. Then the constitutive equations can be expressed as

(1) $$\left(\begin{array}{c} Q\\ \Delta \end{array}\right)=\left[\begin{array}{cc} C & nd_{33}\\ nd_{33} & 1/K_{a} \end{array}\right]\left(\begin{array}{c} V\\ F \end{array}\right)\;$$

In this equation, *Q* is the electric charge on the electrodes of the transducer and Δ the total extension. *V* is the voltage between the electrodes of the *n* sheets of the transducer, *F* is the total force, *C* the capacitance of the PZT transducer without external load (F=0), $K_{a}$ the stiffness of the transducer for *V* = 0, and $d_{33}$ is the piezoelectric charge coefficient assuming that the electric field is parallel to the poling direction of the PZT transducer.

This equation relates the electrical and mechanical field in a PZT transducer and expresses both the direct piezoelectric effect (conversion of mechanical stress into electricity) for the PZT transducer working in sensing mode and the inverse piezoelectric effect (opposite conversion) for the PZT transducer working in acting mode. In sensing mode, *V* as a consequence of the mechanical effect on a deformation Δ or a force F on the PZT transducer is measured. This effect can also be used in energy harvesters by storing the electrical energy. In acting mode, Δ, F is the mechanical effect as a consequence of an applied *V* between terminals. In spite of the simple form of Equation (1), the modelling of a PZT sensor or actuator in a general case is a complex thing, because there are some factors that will affect the parameters of Equation (1).

In the case of submerged PZT transducers there are many factors that convert these equations in complex and sometimes non-linear equations. For example, as shown by Ting [4], with high hydrostatic pressures (deep submergence) the piezoelectric charge coefficient $d_{33}$ is slightly reduced (other piezoelectric coefficients such as $d_{3,1}$ [5] or $d_{3,2}$ [4] may be drastically reduced exhibiting an hysteresis effect). Also a slight dependence of these coefficients with the temperature is shown in the same study. Furthermore, high mechanical stresses associated to high hydrostatic pressures could lead to a failure of the PZT material as reported by Wang and Liu [6]. All these effects have to be considered in deep submerged PZT transducers, such as hydrophones used in oceanic environments for underwater acoustic communications [7]. In this review paper, we mainly focus on applications where the hydrostatic pressure is low and thin PZT transducers (also known as PZT patches) are used independently or attached to the submerged structure. In such cases, there are also many boundary conditions that make the practical resolution of Equation (1) difficult.

Firstly, if we consider that the PZTp attached to a structure is surrounded by air or vacuum, the influence of the surrounding fluid is neglected as a simplification. Considering that the structure is submitted to a static force or a voltage difference is applied between electrodes, the relationship between the total extension of the transducer and the voltage between electrodes has to include characteristics of the structure such as thickness or Young modulus, which will modify the value of $K_{a}$. Furthermore, if the PZTp is used in dynamic mode, non-linear effects, such as hysteresis, have to be considered [1]. Finally, when a structure vibrates in a heavy fluid, as it is the case considered in this paper, the fluid effects are not negligible and have to be included in the dynamic equations. This usually requires considering complex Fluid Structure Interactions phenomena that hinder the resolution of the dynamic problem. In this review paper we will not discuss the complex theoretical or numerical resolution of the dynamic problem of a PZTp attached to a submerged structure, which is very extensive and laborious. Particular solutions of this problem can be found in some of the references presented here.

## 3. Applications of PZT Patches in Submerged Systems

In this section, we discuss the main applications in submerged systems, where PZTp are typically used. As it will be shown, the state of the art of these applications has improved due to the use of PZTp. In fact, PZTp are by far the most used transducers (sensors & actuators) in the reviewed applications in submerged systems. Recent researches, experimental methods and procedures used, and main achievements and results obtained will be discussed for every application.

### 3.1. Modal Analysis of Submerged Structures

Modal Analysis is a widely used technique in many engineering applications, which models a mechanical structure as an assembly of masses, springs, and dampers [8,9,10,11]. One of the main outputs of Modal Analysis is to describe the relationship between the response of the structure (in terms of vibrations) and the dynamic force in the frequency domain, which is known as the Frequency Response Function (FRF). An accurate knowledge of the FRF is of paramount importance to extend the useful life of structures avoiding undesired noise/vibrations, fatigue damages, and resonance problems. Experimental Modal Analysis is based on the excitation of the structure by a known force characteristic (calibrated actuator) and the measurement of its response (sensor).

The FRF can be defined as the superposition of the contribution of N vibration modes. Its generic equation for proportionally damped systems can be expressed as

(2) $$\left[H\left(j\omega \right)\right]=\overset{N}{\underset{r=1}{\sum }}\frac{j2\omega_{r}Q_{r}{\left\{\vartheta \right\}}_{r}{\left\{\vartheta \right\}}_{r}{}^{t}}{\left(\theta_{r}^{2}+\omega_{r}{}^{2}-\omega^{2}\right)-2\theta_{r}j\omega }$$

This equation shows that the FRF, defined in the frequency domain jω, depends on the following modal parameters, which have a special relevance by themselves. These are the natural frequency of the corresponding mode $\omega_{r}$, the mode shape ${\left\{\vartheta \right\}}_{r}$ which defines the deformation shape close to resonance condition ($\omega =\omega_{r}$), the damping ratio $\theta_{r}$ which determines the amplitude of the FRF in resonance condition, and the scaling factor $Q_{r}$ which has an influence on the amplitude of the mode. Due to the importance of modal parameters, many studies are focused on the study and analysis of one of these parameters instead of considering the whole FRF.

Interest in determining the modal parameters in submerged structures can be found in many engineering applications, such as hydraulic turbomachinery, the marine and naval industry, energy harvesting, and micro applications. In order to determine the modal parameters and the FRF, calibrated sensors and force actuators have to be used. In submerged structures, it can be extremely complex to use classical force actuators (impact hammer or shaker), as these structures are many times inaccessible and sometimes confined in small spaces. Furthermore, electronics of the sensors have to be protected from water. As PZTp are small, thin, and light actuators (compared to other electromechanical actuators), which do not interfere in the fluid structure interaction, they have been extensively used as exciters in modal analysis of submerged structures. As a consequence, modal parameters of different types of submerged structures have been experimentally obtained in many complex flow situations.

For more than one hundred years it has been analytically and experimentally demonstrated in simple structures and with simple boundary conditions, that when a structure is submerged in a heavy fluid, such as water, its modal parameters are drastically affected compared to those with air [12,13,14,15,16]. In particular, it has been shown that due to the added mass effect the natural frequencies of submerged structures are generally reduced with respect to those of the structures vibrating in air. From an analytical perspective it is also clear that when the submerged structure is close to a rigid wall, the added mass effect is even increased and the natural frequencies reduced [17,18]. Regarding the mode shapes, it seems that the effect of a heavy fluid slightly modifies them and therefore in many studies this effect is even not considered. For the damping ratio, it is clear that water will contribute to the dissipative effects that will increase the total damping of the vibrating structure. Although these generic conclusions have been analytically obtained before the use of PZTp in submerged structures, most of these conclusions were limited to simplified structures vibrating in inviscid fluids and with simplified boundary conditions.

**Figure 1:**
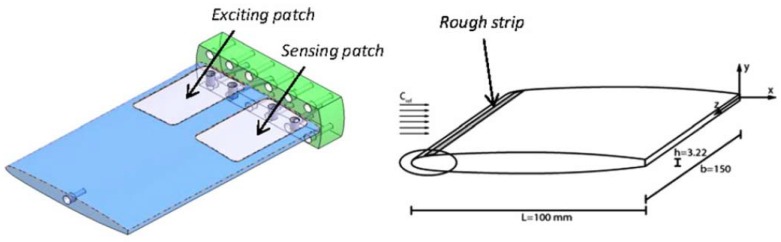
Piezoelectric (PZT) patches installed in a cavitating hydrofoil in order to obtain its natural frequencies [22].

The most relevant modal parameters are probably the natural frequencies. Valentin et al. [19] obtained the natural frequencies of a submerged disk with very small axial and radial confinements to rigid walls by using PZTp. Conclusions regarding the reduction of the natural frequencies with small distances to rigid surfaces were consistent with past analytical models [18]. The case of a flexible disk approaching a non-rigid surface has also been experimentally analyzed by the same author using PZTp [20]. This study shows that the trend of the natural frequencies, when the disk approaches a non-rigid surface is to increase with decreasing distance, which is the opposite behavior to when the disk approaches a rigid surface. A similar phenomenon is observed by Presas et al. [21], when analyzing the natural frequencies of an installed reduced scale model pump-turbine runner. In that case, PZTp are used to determine the Natural Frequencies and mode shapes of the turbine runner in a more realistic boundary condition (submerged and confined). At present, according to the authors’ knowledge, there are not satisfactory analytical or numerical models that can describe the influence on the natural frequencies of nearby non-rigid surfaces. In this context, PZTp have contributed to obtain reliable experimental results. Another complex flow situation, where PZTp have been successfully used, is the analysis of the natural frequencies of cavitating structures. In such case, it is expected that the added mass effect will be less important due to the presence of air. De La Torre et al. [22] used PZTp to obtain the natural frequencies of a submerged hydrofoil with different incident speeds and cavitation conditions. In that case, the hydrofoil is rectified (special cavities of 0.35 mm for the PZTp are manufactured) in order to attach two PZTp flush mounted to the surface. In that experiment, the use of PZTp permitted obtaining the natural frequencies of the hydrofoil without disturbing the incident flow. One PZTp is used for exciting and the other for measuring the response (Figure 1). Results show that with respect to the non-cavitating case, the natural frequencies tend to increase with increasing cavitation volume near the hydrofoil.

**Figure 2:**
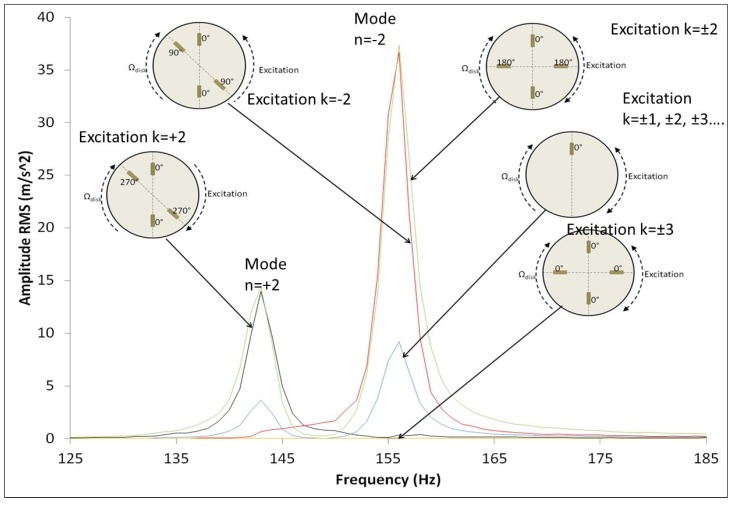
The excitation of the rotating mode shapes of a rotating submerged disk by means of PZT patches [26].

PZTp have also been used to experimentally obtain, for the first time, the natural frequencies of a rotating disk in water, which has a relevant interest in Hydraulic Turbomachinery. Presas et al. [23,24,25] proved experimentally the analytical model proposed by Kubota [17]. In these studies, PZTp are used as exciters of the rotating disk. Regarding the natural frequencies and mode shapes it is shown that when the disk rotates surrounded by a heavy fluid every natural frequency is separated in two natural frequencies and the mode shapes become rotating mode shapes (travelling modes), even for very low velocities. The lower mode rotates in the same direction as the disk and the higher in the opposite. The same author in [26] used several PZTp to simulate the response of a disk-like structure due to real excitation patterns occurring in hydraulic turbines (Rotor Stator Interaction). Particularly it is shown that in order to have a resonance, the excitation shape has to be directional and rotate in the same direction as the corresponding mode shape. This is shown in Figure 2, where it can be seen that resonance only occurs if the excitation has the same pattern as the mode shape. As seen in this figure, the excitation pattern is generated with simultaneous PZTp working with different phases.

The use of PZTp in submerged structures has also permitted determining the damping ratios of standing disks and analyzing the influence of nearby rigid walls on them. In the work of Valentin et al. [19] it is shown that damping ratios increase as the distance between the disk and a rigid wall decreases, which is attributed to higher dissipation effects, as the water has to flow through very small gaps. Presas et al. [27] determined the damping ratio for the first several modes of a submerged disk and compared them to the ratios obtained with a classical excitation method (impulse hammer). An interesting conclusion is that when the disk is in infinite water medium both methods give the same damping ratios, which also validates the use of PZTp to obtain them. Nevertheless, when the disk is close to a rigid wall, the use of an impulse method (instrumented hammer) overestimates the damping ratio, as the impulse causes a motion of the disk towards the wall, affecting the results.

Although PZTp have been shown as suitable exciters to obtain most of the modal parameters in submerged structures, the main drawback compared to a calibrated exciter (such as an instrumented hammer) is that there is not a linear relationship between the voltage applied and the force exerted on the structure. Therefore, an FRF in terms of displacement/force (m/N) (also velocity/force and acceleration/force) which is the FRF used in modal analysis, cannot be directly obtained. Instead, a transfer function between displacement and voltage of the signal applied (m/V) or (V/V) is generally given. In the work of Presas et al. [27], it is proposed to introduce the natural frequencies, mode shapes and damping ratios, experimentally obtained with PZTp, in a Finite Element Simulation model. In this way, it is shown that the FRF used in modal analysis (m/N) can be obtained.

### 3.2. Active Vibration Control (AVC) of Submerged Structures

**Figure 3:**
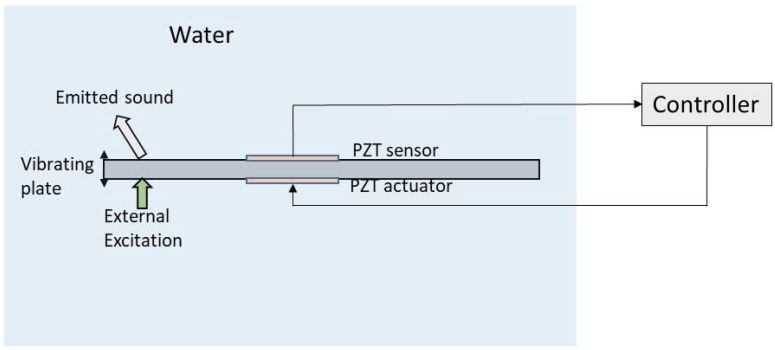
Active vibration control with a PZT sensor and PZT actuator.

Active vibration control consists in reducing the amplitude of the dynamic response of a mechanical structure that is being excited by an external force. In order to suppress vibrations, a sensor and an actuator are needed. The signal of the sensor is used as input of an analogical or digital controller, which generates a signal for the actuator in order to suppress the existing vibration [28,29,30]. In the recent years, PZTp have been widely used as sensors/actuators for active vibration control [31,32] and particularly also for submerged structures, as they are easy to install, they do not affect the existing flow and they can be easily insulated with an epoxy layer. Besides PZTp, mechanisms based on eddy currents (Eddy Current Tuned Mass Damper [33]) and based on mechanical tuned dampers (Pounding Tuned Mass Damper [34]) have been recently investigated to suppress vibration in submerged pipe lines. The scheme representing the idea of active control of vibrations in a submerged structure is represented in Figure 3. It is worth mentioning that in this subsection we analyze studies dealing with active vibration control, that is, the reduction of vibration of submerged structures. As a consequence, the emitted sound is also attenuated, although it is not the primary objective of the control. In the next subsection, we focus on the cancellation of the emitted sound of an acoustic surface, from an arbitrary source location and not necessarily the vibration of the surface (Figure 5).

The key point in active vibration control is precisely the controller design. From the theoretical point of view, the controller has to be designed considering the structural equations, the coupled fluid/structure interaction and the constitutive equations of the PZT sensor/actuator. Due to its similarity with real engineering applications, mainly two types of structures have been considered to address the topic from the theoretical, numerical, and experimental points of view. These structures are thin plates and cylindrical shells. The literature existing for PZTp used for active vibration control of structures vibrating in air, where the effects of the surrounding fluid are negligible, is extensive (see for example [35,36,37] for plates and [38,39,40] for cylindrical shells). The case of submerged structures, which is more complex as it has to include the fluid–structure interaction in the design of the controller, has been less analyzed.

**Figure 4:**
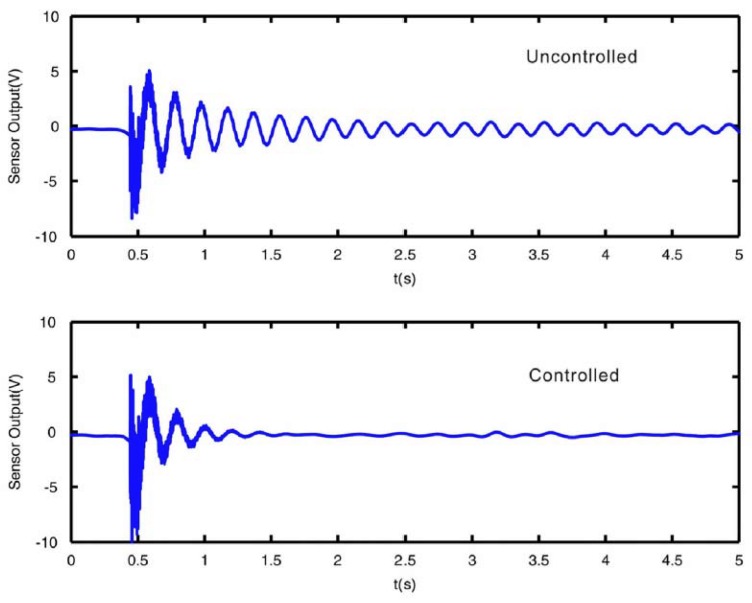
Response of a partially submerged plate due to an impact measured with a PZT patch. Uncontrolled and Controlled system (by means of a PZT patch actuator) [44].

Regarding submerged thin plates, Li [41,42] simulated the active control of a fluid-loaded plate using PZT sensors/actuators. In [41] the plate is modeled by a finite element formulation taking into account the Fluid Structure Interaction. A negative velocity feedback control algorithm is used, showing that damping of the structure can be actively increased. A different approach for the controller design is followed in [42]. In this case, the modal approach, that is, the decomposition of the dynamic equations according to modal analysis techniques (see Section 3.1), is used to analyze the same problem. According to the author, this approach has the advantage of being simpler, compact and physically interpretable. Results from this study show that the modal controller can act on a particular mode shape by increasing the modal damping. Some studies include not only a theoretical modelling of the design of the controller, but also experimental validation. Kwak et al. [43,44], modelled the structure, the fluid effect, and the constitutive equations of PZTp, in a partially submerged plate equipped with PZT sensors and actuators to design the controller and validate the model with experimentation. In this case a MIMO (Multiple-Input Multiple-Output) digital controller is designed (two PZT actuators and two PZT sensors are used). An example of the damping effect of the active vibration control in the submerged plate is shown in Figure 4 [44]. In both cases, the plate is excited with an impact of similar force (external excitation) and the responses of the structure with and without active control are represented. It can be seen that the actuator quickly reacts after the first impact and is able to suppress the free vibration in a short time.

Regarding submerged cylindrical shells, Pan et al. [45] numerically analyzed the reduction of the radiated pressure of a cylindrical shell type structure (submarine hull) by reducing the structural vibrations in the axial direction (low frequency modes). In both cases, PZTp are circumferentially mounted. The designed control system in [45] is capable of reducing the radiated pressure by than half for the three first axial modes. The work of Kwak et al. [46], theoretically models the active vibration control of submerged cylindrical shells. This work is also validated with an experimental test rig. The active vibration control is tested for a harmonic disturbance, showing that the controller can suppress the vibration having either a resonant or non-resonant frequency. This study also shows that by reducing the vibrations of the cylindrical shell, the radiated sound is decreased, which is also experimentally confirmed in the work of Loghmani et al. [47].

### 3.3. Sound Cancellation of Submerged Systems

**Figure 5:**
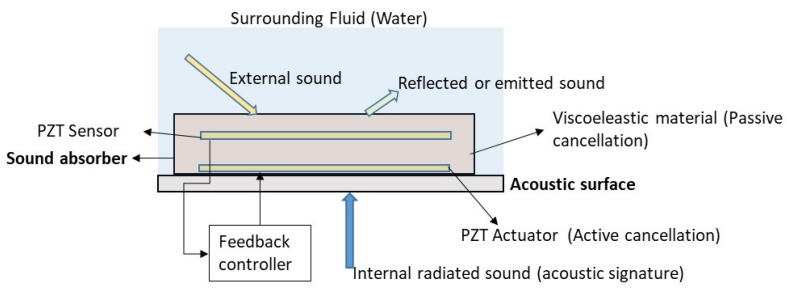
External, internal and reflected sound in an acoustic surface. Active/passive sound cancellation techniques.

Besides the vibration control of submerged structures, the other interesting problem, where active control is required, is the reduction of the sound that is emitted by a submerged acoustic surface. Considering that the surface is an external acoustic surface of a system (biological or mechanical), the sound can be radiated from an external source and reflected on the acoustic surface or can be internally originated in the system, which is known as the acoustic signature of the system (Figure 5). The acoustic signature is the combination of all the acoustic and sounds emissions of a system (device, mechanism, vehicle, living organism, …). Typical submerged systems are ships, submarines [48,49], and also living organisms [50,51]. It is well-known that the detection of the acoustic signature with remote sensors is a field of interest for military purposes [52,53] and also in the study of marine species [50] as it can give information of the type, state, and position of the considered system. In the military field the main interest focuses on the reduction of the emitted sound originated internally (acoustic signature) in ships and submarines and also in the reduction of the reflected sound (Figure 5), with the purpose of making the system “acoustic” invisible to radars and detecting systems. Therefore, different passive and active noise cancellation (ANC) techniques have been developed in the past.

The first and simplest approach is the passive cancellation of sound. The technique consists in attaching viscoelastic materials to the acoustic surfaces where the sound is emitted (internal sound or reflected sound) [54,55,56]. The basic principle is to induce cyclic strain energy losses in the viscoeleastic material [57]. Therefore, optimal locations are points with maximum strain energy which can be obtained with Finite Element simulations [58]. To address the problem, some researchers model the acoustic absorber and the surface as an impedance [54,59,60]. The problem then consists in an optimization of the impedance of the absorber (which mainly depends on the thickness) to maximize the sound absorption.

**Figure 6:**
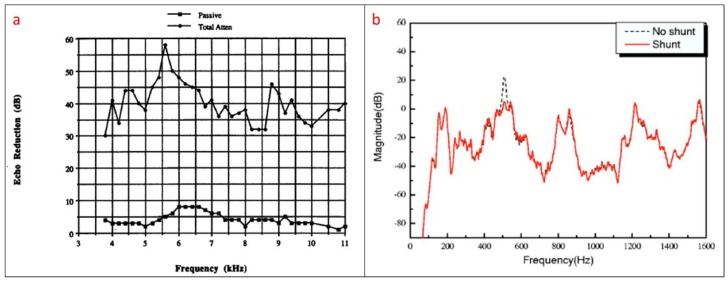
(**a**) Passive/active sound absorber in underwater. Reduction of noise for a broad bandwidth for passive mechanism and passive/active actuator [61]; (**b**) Reduction of noise for a particular mode with shunt method [67].

The effectiveness of the sound absorber is greatly improved if active techniques, combined with passive absorbers are used. This is the second type of approach which is usually called passive/active absorber. The idea of the active cancellation is to detect the sound close to the acoustic surface with an adequate sensor. The signal of the received sound is then used in a feedback system that controls an actuator which tries to attenuate the sound in real time by generating a destructive signal that opposes the surrounding noise. For this purpose, in many cases sensing/acting PZTp have been used in passive/active sound absorbers [52,54,55,56,57,61,62,63,64,65]. A schematic of a passive/active sound absorber scheme is shown in Figure 5. In sound cancellation the acoustic pressure has to be measured with an appropriate sensor. If the acoustic pressure is measured with sensors bonded on the surface (such as represented in Figure 5), then the technique is normally known as Active Structural Acoustic Control (ASAC) while the term Active Noise Control (ANC) is normally used for acoustic pressure obtained with microphones [63]. One of the first works related to active cancellation of sound by means of PZT transducers is the research of Braga et al. [66]. They show that, for a piezoelectric plate submerged in a fluid, it is possible to cancel the reflected sound of the surface for a given frequency and angle of incidence. Although this work is focused on the cancellation of reflected noise, it also suggested that the cancellation of the radiated internal sound (acoustic signature) would be also possible (Figure 5). The work of Howarth et al. [61] introduces the concept of “piezoelectric coating” for submerged structures. This study proposes an active coating consisting of a sensor which controls the feedback mechanism of the actuator, which is used to cancel the sound. This “active coating” cancels the reflected sound along all the acoustic boundary. With respect to the passive method, the passive/active method improves the performance of the cancellation [52]. Similar techniques of passive/active cancellation of noise have been used in problems related to flow ducts [67]. Figure 6a shows the difference in noise reduction for a passive/active sound absorber for the condition that only the passive material is acting and when the active system is functioning. The results correspond to a passive/active sound absorber submerged in water and working with PZT transucers (sensor and actuator).

Another approach which has been recently used in sound absorption of submerged structures is the use of shunt impedances [67,68,69,70]. The basic idea of such method is that the piezoelectric layer coating of the acoustic surface is associated to an R-L-C circuit. The values R-L-C are selected so that the absorber can absorb sound energy at one particular frequency with some bandwidth around it. This technique has also been called in some references semi-active control [68]. With respect to the active control, in this case, the mechanical energy produced by the sound is converted into electric energy through the piezoelectric effect and dissipated in the shunt circuit. This method has also the advantage that a PZT coating with shunted circuits is simpler, stable, cheaper than a passive/active device [68]. Nevertheless, with respect to the active control, where a large bandwidth is attenuated (Figure 6a), with this technique, when using only one shunt circuit, only one single mode (narrow frequency band) can be attenuated (Figure 6b) as the device can only absorb sound energy in a narrow band, depending on the selected values R-L-C. Therefore, in order to extend the attenuation bandwidth associations of more than one circuit (every circuit tuned for one particular frequency and bandwidth) can be used (multimode shunt damping [70]). Also, several works have been carried out in order to optimize the bandwidth of every shunt circuit. The work of Zhang et al. [69] concludes that when shunting a PZT transducer with only one resistor and inductor, only a narrow bandwidth of sound can be absorbed. In order to increase this bandwidth, using a resistor and a capacitor is proposed.

### 3.4. Atomic Force Microscope (AFM) in Physiological Mediums

One application where PZT transducers are fundamental is in an Atomic Force Microscope (AFM). The AFM, which was invented more than thirty years ago [71,72], permits the exploration of the topography of surfaces in nanoscale and measurement of its superficial forces. This has several applications in fields such as solid-state physics, semiconductors, and to investigate physiologic samples in small scales, which involves research related to biology and medicine [73].

**Figure 7:**
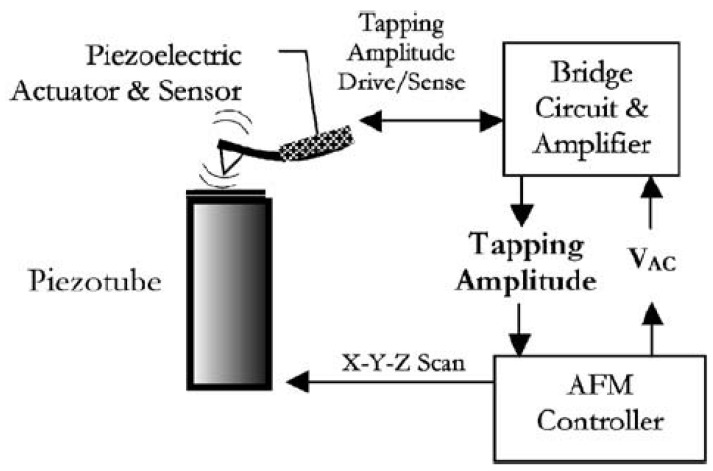
Schematic of an Atomic Force Microscope (AFM) with a piezoelectric sensor and actuator [82].

The AFM consists of a microcantilever (size of few µm [74]) with a sharp tip at the end. The tip, which has a curvature radius of few tens of nanometers, scans the surface of the sample (see scheme of Figure 7). Different operating modes can be distinguished in AFM depending on the relative motion of the tip with respect to the sample (scanning mode). Historically, the first mode used was the contact mode (static mode) where the tip is dragged over the sample. The small deflections of the tip, which are related to the surface topography and intermolecular forces, are recorded by a sensor. In this mode, the lateral forces tip-surface may be important as the contact of the tip-surface is permanent. This implies more failures of the tip and of the sample. The tapping mode or vibrating mode partially avoids this problem as the lateral forces are much smaller and the contact tip sample is intermittent and lasts few µs. In this mode, the cantilever is forced to vibrate close to one of its natural frequencies.

In many cases, in order to excite the cantilever, PZT transducers are used, although other options may be possible. The PZT actuator can be attached on the cantilever holder, but also cantilevers fully made of piezoelectric materials have been widely used [75,76]. The amplitude of the oscillating cantilever is in range of several nanometers [77] and kept constant in the tapping mode, sometimes by means of a feedback system. Therefore, an AFM image of the sampled surface is obtained by means of intermittent contact between the tip and the surface [78].

In both modes, the deflection of the cantilever due to the contact to the surface has to be measured. Although the traditional method is the beam deflection method by means of a Solid State Laser Diode, more recently, this measurement has been performed by means of a PZT sensor showing similar accuracy. As pointed out in many research works, the use of PZT transducers in lieu of other sensors permits achieving higher scanning speeds [79,80,81,82,83] and much more compact systems that avoid the problem of aligning the laser beam and refractions in submerged samples [82,84,85]. The complete scheme of an AFM working with an integrated PZT actuator and sensor is shown in Figure 7.

The “tapping mode” is the most commonly used mode in biological and medicine research of physiologic samples, where the normal condition of such samples is submerged in fluid mediums. Working the AFM in this mode, the surface of fragile organic samples (such as DNA specimens) are more protected from the potential damages caused by the cantilever tip [86,87]. Furthermore, this mode allows much higher scan speeds [79], which is of paramount importance when analyzing some biological processes that occur in few seconds. Recently developed modes for biological applications and combined modes with TIRFM (Total Internal Reflection Fluorescence Microscope) are discussed and reviewed in [88,89]. The scanning speed of the AFM in the “tapping mode” depends on the resonant frequency of the cantilever, as in the tapping mode the cantilever is driven at this frequency. As mentioned in Section 3.1, it is well-known that natural frequencies of submerged structures in heavy fluids are greatly reduced with respect to the vacuum case (or air) and therefore related to AFM, several studies considering the dynamic behavior of submerged microcantilevers made with PZT materials or equipped with PZT cantilevers have been performed.

**Figure 8:**
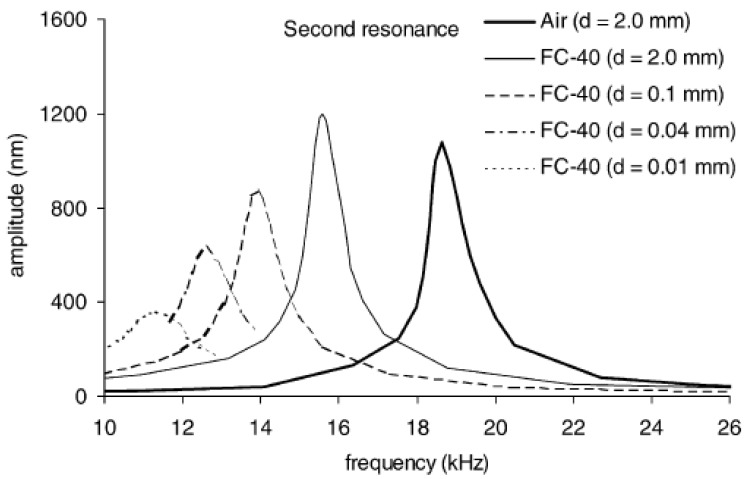
Influence of the cantilever–surface position on the resonant frequency [92].

The works of Sader [90] and Green & Sader [91] provide theoretical models of the vibrating frequencies and modes of submerged microcantilevers. Sader [90] pointed out that analytical and numerical models used for larger structures normally consider the fluid as inviscid. Neglecting the viscosity of the fluid and the damping effects is a reasonable approach for macrostructures in order to predict the position of the resonant peaks, but in microscales damping effects greatly modify the position of the resonant peak and therefore viscosity effects have to be considered. In this work, Sader presents a theoretical model to determine the Frequency Response Function of submerged cantilevers considering an incompressible flow with viscosity. One of the most interesting conclusions is that when the dimensions of the beam are reduced, the viscous effects become more important and therefore the natural frequencies are more reduced with respect to those values in vacuum. Regarding the proximity of surfaces, Green [91] found that for distances larger than the width of the cantilever, the cantilever can be assumed to be immersed in infinite fluid. For smaller distances, strong dissipative effects that decrease the frequency and amplitude of the resonant peaks are observed. One of the research works where these effects are experimentally demonstrated is the work performed by Naik et al. [92]. As seen in Figure 8, strong dissipative effects greatly modify the shape and amplitude of the peaks when the cantilever is close to a rigid surface, due to an increased damping. The influence of the mass and damping of the tip (Figure 7) on the vibration characteristic of the AFM probe, which is not considered in [92], has been recently analyzed by Korayem and Nahavandi [93].

The other point to be considered when working with submerged PZTp is the coating and isolation of the piezoelectric material from the fluid. The research of Rogers et al. [81,82] provides a good comparison between different options. Particularly in [81] three different coating options were tested. Fluoropel (a 1% fluoropolymer solution in fluorosolvent) did not show satisfactory coating properties, especially when applying 10 V DC. Using Parylene (conformal coating) 10 of 16 cantilevers coated with this protection functioned normally after applying 10 V DC. Nevertheless, Parylene was proven to be unsatisfactory for the wires connected to the PZTp and caused a lot of troubles when trying to add another material, such as two stage epoxy. The best coating solution, according to that study, is a silicone-nitride film over the cantilever surface, which has shown durability in water and saline under normal working conditions.

### 3.5. Energy Harvesting

One of the most relevant and growing field in the recent years is energy harvesting. The evolution of energy harvesting applications has grown together with the evolution of PZTp in the last two decades [94,95,96,97]. The main interest of this topic is to fabricate wireless autonomous sensors in remote locations avoiding battery replacements and charges. It is well known that other sources, such as solar energy, can provide a power density of 15,000 µW/cm^3^ in directly radiated objects, although this energy is not usable in non-directly radiated surfaces (indoor applications), as the density will drop to 10–20 µW/cm^3^. In such situations, the conversion of mechanical vibrations into electric energy can provide around 300 µW/cm^3^. In this context, PZTp are preferred before other electromechanical transducers (see [98] for these type of harvesters), due to their efficiency and reliability [99,100,101]. Energy Harvesters used in submerged applications based on PZTp have been developed in the last decade as pointed out in many studies. The idea is to use the energy of flowing water or pressure oscillations in closed systems in order to power self-sustainable wireless sensors in oceanic [102], biological [103], and health monitoring sensors [104].

**Figure 9:**
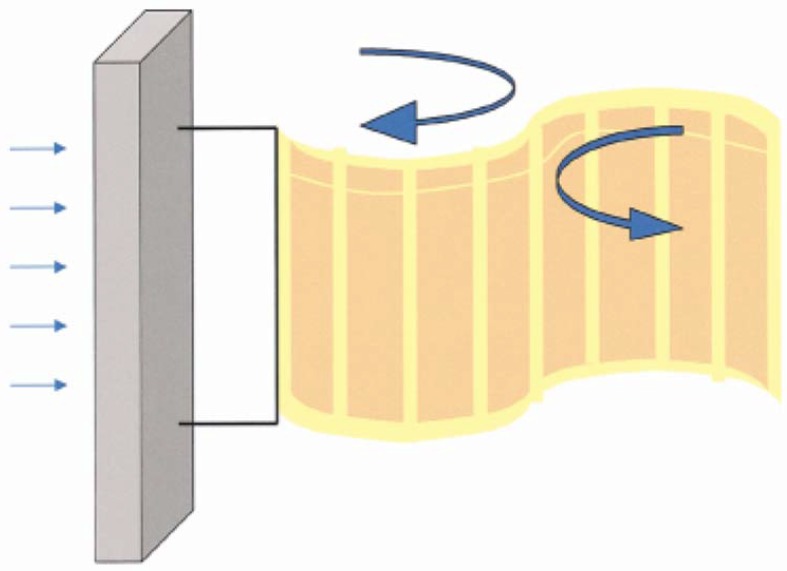
Oscillating eel (piezoelectric membrane) behind a bluff body [106].

One of the first studies which analyzes the feasibility of obtaining energy by means of a flowing water current is the work made by Allen and Smits [105]. The basic idea is to place a piezoelectric membrane (called “eel”) in the wake of a bluff body and use the Von Karman street vortices that force the eel to vibrate. This study, which is focused on the flow analysis around the eel, shows that under certain flow conditions, the piezoelectric eel can exhibit a lock-in behavior and consequently large deformations. These deformations can be turned into electricity power by means of the piezoelectric effect. A scheme of a piezoelectric eel, placed behind a bluff body, is shown in Figure 9. The work of Taylor et al. [106] followed the idea of the eel and developed a prototype for extracting energy of flowing water in rivers and oceans. They use piezoelectric polymers (PVDF) for this purpose. A complete analysis of all the steps of the design—flow analysis (similar as in [105]), piezoelectric material, shape, and an optimized resonant electric circuit—is provided in this study. The optimization of the electric resonant circuit in energy harvesters is also a complex problem. Similar as discussed in Section 3.3 (semi-active sound cancellation), the idea is to determine the parameters R-L-C, where the device harvests energy in a more efficient way. Similar eel mechanisms have been also analyzed in similar studies and patents [107,108].

Further energy harvesters using the energy of free water flowing around a body have also been developed. Song et al. [109] proposed an energy harvester based on the vortex-induced-vibration (VIV) of a cylinder flowing in fluid. In such a mechanism, the cylinder is partially submerged in flowing water. The cylinder is joined with a beam where the PZTp is attached. VIV bends the cylinder and the shaft in a cyclical way. This mechanical energy is transformed into electrical energy by means of the piezoelectric transducer. This device has been experimentally tested in the laboratory in a water canal. An interesting conclusion for the design of the harvester is that reducing the mass of the cylinder and increasing the diameter increases the ability to harvest energy. The idea of a partially submerged cantilevered structure is also investigated by Cha et al. [110]. In this study, which aims to be useful for design of energy harvesters partially submerged, a cantilevered beam is forced to vibrate in a tank (without flowing water). Several geometries and configurations are experimentally tested in that study and interpreted with a nonlinear distributed piezohydroelastic model. Experimental results show that increasing the wet length reduces the resonant frequency, due to added mass (see also Section 3.1) and the harvested energy is also reduced (which is in accordance with [109]). More recent works of the same authors investigate the effect of having an eccentric cylinder [111] and of using two identical cylinders harvesting energy in the same water flow [112]. The same principle used for harvesters working with air flows has been also recently shown by Zhang et al. [113]. Finally, it is worth mentioning the work of Li et al. [114] as it provides an excellent review regarding solutions and design of energy harvesters in applications where the main input frequency is in the range of 0–100 Hz (low frequency), which is the case with the aforementioned studies. This includes a discussion and comparison of types of materials, geometries, techniques to match the resonant frequency and design of electronic circuit in such harvesters, which is a complex optimization problem.

**Figure 10:**
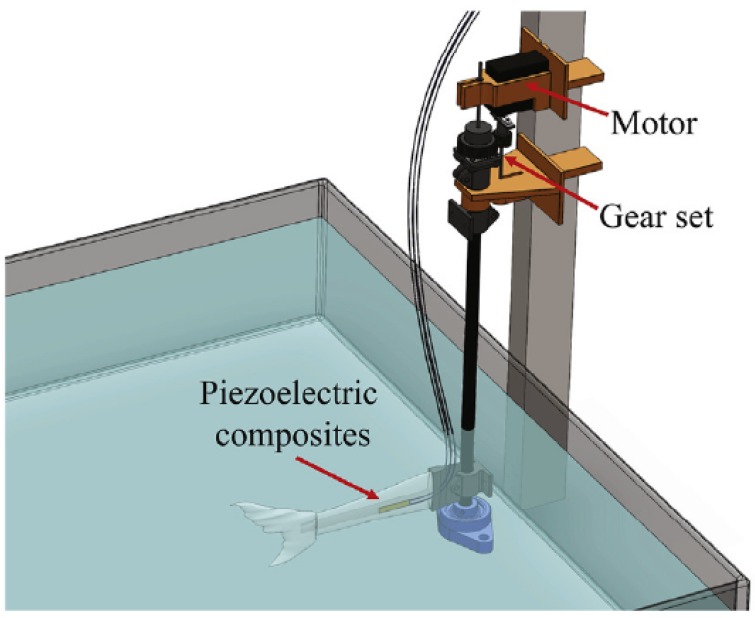
Energy Harvesting in a biomimetic fish [116].

Energy harvesters based on the motion of marine animals and fishes have been recently investigated. The work of Ertkurk and Delporte [115] investigates the energy harvesting in biomimetic or bio-inspired structures (imitating a fish). In this study, macro-fiber composite PZTp are used. They investigate the feasibility of having self-powered swimmer-sensors devices in the future. This is, a device which harvests energy during one phase and then, when enough energy is harvested, this energy is used to move the same device through water. Therefore, they focus not only in the energy harvesting of the PZTp but also in the capacity of the PZTp to generate thrust force. The main conclusion regarding the design is that the presence of a tail fin (similar as in fishes) increases the effectivity of the energy harvesting and thrust generation processes. This is mechanically justified as follows: with the presence of the tail fin, the second structural mode shape approaches towards the first, which results in a wider frequency band for energy harvesting and thrust generation. Understanding the energy harvesting of bio-inspired structures could also have a relevant application in the track of marine species in order to understand migratory patterns. Cha et al. [116] recently investigated the energy harvesting using the motion of a biomimetic fish (Figure 10). Based on the motions of the tail fin (imitating the swim of fishes), they develop a model to predict the energy harvested by two piezoelectric composites. Using this setup, they show the feasibility of monitoring the temperature and transmitting it wireless using only the energy harvested by the tail fin motion. The first energy harvester system (based on PZTp) implanted in a living fish has been reported in [103]. This work proves the feasibility of replacing less efficient and sustainable systems such as acoustic telemetry, which rely on the capacity of the batteries. Furthermore, this implanted PZTp device permits a higher mobility of the tracked fishes, as it does not interfere the natural swim.

All these energy harvesting applications are based on the motion of mechanical or living structures in flowing water. There are a couple of works, which have investigated the feasibility of harvesting energy of the pressure ripples in pressurized water. Wang and Liu [117] develop an energy harvester working in shear mode working with pressurized water flow. The idea is that the pressure ripples can deflect a PZT transducer film attached in a flexible pipe (the flexible part is only a very small part of the total pipe). When the pressure of the inner flow oscillates, the PZT transducer is deformed and thus generates an electrical voltage which can be converted into electrical energy. With the purpose of designing self-powered wireless system in piping systems for health monitoring, Cunefare et al. [104] proposes an energy harvesting system based in the same phenomenon.

Table 1 shows the different harvesting power capacities based on the working principle of the energy harvester. For the harvested power, relevant working conditions, such as velocity or pressure of the flow, are indicated. This means that a change of these working conditions would also change the harvested power. Furthermore, it is also highlighted whether the power is based on predictions of the respective authors or directly experimentally obtained. The size (length, width, and thickness) of the PZT are also indicated. With these two parameters, an approximate value for the power density (W/mm^3^) of each harvester has been calculated, which is very useful to compare the capacity and performance of every type of device.

## 4. Concluding Remarks

Nowadays PZT transducers are extensively used in many engineering, biological, and medical applications. Particularly in submerged systems, PZTp have been widely used as sensors and actuators in many research works. In this paper we have analyzed and reviewed the most typical applications involving submerged systems, such as modal analysis, active vibration control, sound cancellation, AFM, and energy harvesting, where PZTp have been extensively used. Thanks to PZTp, new interesting results, devices, and techniques have been developed for each of these applications. These findings have been carefully analyzed, summarized, and described in this review. Some concluding remarks for each application:

1. ***Modal Analysis of submerged structures:*** By means of PZTp acting as exciters, modal parameters such as natural frequencies, mode shapes, and damping ratios have been experimentally obtained for submerged structures with complex boundary conditions (confined structures, rotating structures, cavitating structures, non-rigid boundaries) for the first time. These exciters are thin, light, and small and do not disturb the flow. The main drawback is that there is not a linear relation between voltage/force and therefore the FRF in terms of displacement/force cannot be easily obtained.
2. ***Active vibration control (AVC) of submerged structures:*** The active vibration control of submerged structures, which has a relevant interest in many engineering applications, has been recently investigated by some researchers. PZTp are almost the only type of sensors/actuators used for this purpose. Numerical and Experimental results performed in thin plates and cylindrical shells show the potentiality of using active vibration control systems based on PZTp to suppress vibrations in generic submerged structures.
3. ***Sound cancellation of submerged systems:*** Passive/active sound absorbers (PZT sensor + PZT actuator + viscoelastic material) have shown good attenuation characteristic in a broad frequency bandwidth, when they have been used in submerged systems. Compared to simple passive systems, based on viscoelastic materials, a much higher effectiveness has been experimentally demonstrated. Semi-active absorbers (PZT layer + shunt circuits) are a simpler and cheaper option, where every shunt circuit is tuned to effectively cancel one frequency band. Therefore, this system may be used in case a finite number of frequencies has to be attenuated.
4. ***Atomic force microscope in physiological mediums:*** The use of PZT transducers over other sensors & actuators used in AFM, considerably simplifies the device. Furthermore, when the AFM works in tapping mode scanning physiological samples in submerged mediums, the use of PZT transducers allows very high scanning speeds which is of paramount importance to observe biological processes on-live and also protect the fragile surfaces of the samples from the scanning forces.
5. ***Energy harvesting:*** In recent years, PZTp have been widely used for energy harvesting in submerged applications. This has permitted the design of wireless and self-sustainable sensors that do not use batteries (and are therefore more ecological) for oceanic, biological, and health monitoring applications. In the present work, different ways to harvest energy from water currents by means of PZTp, which have been recently used, have been reviewed.

## Figures and Tables

**Table 1:** Approximated power, approximated size of the PZTp and power density (W/mm^3^) based on the different working principles.

**Eel**	Taylor et al. [106]	1 W (Predicted at 1 m/s) 1320 mm × 152 mm × 0.4 mm (5 PZTp) **2.5** × **10^−6^ W/mm^3^**		
**Cantilever with cylinder partially submerged**	Song et al. [109]	85 µW (v = 0.35 m/s) 70 mm × 20 mm × 0.2 mm **3** × **10^−8^ W/mm^3^**	Shan et al. [111]	400 µW (v = 0.35 m/s, eccentric cylinder) 85 mm × 25 mm × 0.2 mm **9** × **10^−8^ W/mm^3^**
**Pressure Fluctuations**	Cunefare et al. [104]	1.2 mW (Predicted, Mode 33, 400 kPa) 6.8 mm × 6.8 mm × 30 mm **8** × **10^−7^ W/mm^3^**	Wang et al. [117]	4.9 µW (Predicted, Mode 15, 400 kPa) 8 mm × 3 mm × 0.2 mm **1** × **10^−6^ W/mm^3^**
**Fish Tail**	Li et al. [103]	24 µW (Robotic fish, bending radius 10 cm) 10 mm × 3 mm × 0.2 mm **4** × **10^−6^ W/mm^3^**		
**Quiescent Water**	Cha et al. [110]	10 µW (Predicted at resonant frequency) 102 mm × 35 mm × 0.2 mm (2 PZTp) **7** × **10^−^^9^** **W/mm^3^**		
